# Identification of Wild Boar–Habitat Epidemiologic Cycle in African Swine Fever Epizootic

**DOI:** 10.3201/eid2404.172127

**Published:** 2018-04

**Authors:** Erika Chenais, Karl Ståhl, Vittorio Guberti, Klaus Depner

**Affiliations:** National Veterinary Institute, Uppsala, Sweden (E. Chenais, K. Ståhl);; National Institute for Environmental Protection and Research, Rome, Italy (V. Guberti);; Friedrich Loeffler Institute, Greifswald-Insel Riems, Germany (K. Depner)

**Keywords:** African swine fever, ASF, wild boar, habitat, carcass, transmission cycle, epidemiologic, epizootic, sylvatic, tick–pig, soft tick, domestic, *Ornithodoros*, tick, parasite, warthog, domestic pig, anthropogenic, sub-Saharan Africa, Iberian Peninsula, Eurasia, European Union, Caucasus, Moldova, Romania, the Russian Federation, Ukraine, Baltic States, vector-born infections, viruses

## Abstract

The African swine fever epizootic in central and eastern European Union member states has a newly identified component involving virus transmission by wild boar and virus survival in the environment. Insights led to an update of the 3 accepted African swine fever transmission models to include a fourth cycle: wild boar–habitat.

The main components in the epidemiology of African swine fever (ASF) have been known since the first description of the disease: soft *Ornithodoros* spp. ticks, warthogs, domestic pigs, and pig-derived products such as pork. Three independent epidemiologic cycles (sylvatic, tick–pig, and domestic) have been described (*1*) (Figure). In the sylvatic cycle, ASF virus circulates between the natural reservoirs of the virus (i.e., warthogs and soft ticks), without causing disease in the warthogs. This ancient cycle is the origin of the tick–pig cycle and the domestic cycle and thus the origin of ASF as a disease. In the tick–pig cycle, the virus circulates between soft ticks and domestic pigs. This cycle has mainly been described in sub-Saharan Africa, but also played an important role during the epizootic on the Iberian Peninsula. In the domestic cycle, the virus is transmitted among domestic pigs, or from pig products to domestic pigs. This cycle does not involve the natural reservoirs.

**Figure:**
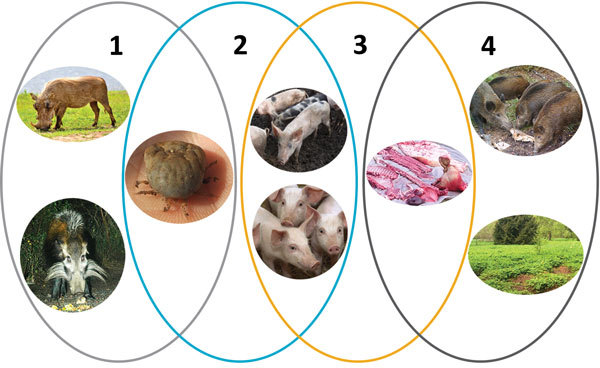
The 4 epidemiologic cycles of African swine fever and main transmission agents. 1) Sylvatic cycle: the common warthog (*Phacochoerus africanuus*), bushpig (*Potamochoerus larvatus*), and soft ticks of *Ornithodoros* spp. The role of the bushpig in the sylvatic cycle remains unclear. 2) The tick–pig cycle: soft ticks and domestic pigs (*Sus scrofa domesticus*). 3) The domestic cycle: domestic pigs and pig-derived products (pork, blood, fat, lard, bones, bone marrow, hides). 4) The wild boar–habitat cycle: wild boar (*S. scrofa*), pig- and wild boar–derived products and carcasses, and the habitat.

In 2007, ASF was introduced into Georgia in Eurasia. The epizootic was not brought under control, and the disease spread to the surrounding countries, including the Russian Federation, and further to Belarus and Ukraine (*2*). In 2014, ASF reached the European Union (EU) member states of Estonia, Latvia, Lithuania, and Poland; in 2016, Moldova; and in 2017, the Czech Republic and Romania. In the ongoing epizootic in the Caucasus, Moldova, Romania, the Russian Federation, and Ukraine, the epidemiology seems to follow the common domestic cycle: the infection circulates among small pig farms, affecting few commercial farms, and somewhat frequently spills over to wild boar (*3*). A similar cycle has been present in Sardinia since 1978 (*1*). Since 2014, the affected EU member states have applied a common reporting framework and shared outbreak data. From these data, a previously undescribed epidemiologic pattern became evident: a cycle that focuses on the wild boar population and its habitat as a virus reservoir (*4*) (Figure). We suggest naming this cycle the wild boar–habitat cycle.

In the ongoing epizootic, ASF disease dynamics have proven to be complex and difficult to control (*5*). ASF prevalence remains <5%, and a pattern of local persistence with slower than expected dynamic spatial spread is evident, estimated at an average of 1–2 km/month (*6*). During 2016 in the Baltic states, <85% of wild boar found dead were ASF virus–positive, although virus prevalence in hunted wild boar was very low (0.5%–3%) (*6*). Currently, a standardized approach for estimating prevalence is lacking, and depending on which areas (infected, surveillance, or unrestricted zones) and categories (found dead, killed in car accidents, or hunted) of wild boar that are included, the reported figures can underestimate or overestimate the true prevalence. The prevalence of antibody-positive hunted wild boar is lower than the virus prevalence for all infected countries and has no clear temporal trend. The low prevalence in hunted wild boar is to be expected, because this group represents an apparently healthy population, considering the nature of the disease and its high case-fatality rate among wild boar (*7*).

The wild boar–habitat cycle is characterized by both direct transmission between infected and susceptible wild boar and indirect transmission through carcasses in the habitat. The habitat contamination from ASF virus–positive wild boar carcasses, and the possible subsequent intraspecies scavenging (*8*), offer possibilities for both low-dose and high-dose infections, depending on landscape, time, season, and carcass decomposition. These epidemiologic drivers of disease intermingle with wild boar population determinants such as wild boar demography, including fertility; management factors such as winter feeding to avoid wild boar population crashes associated with cold weather and feed scarcity; hunting rates; hunting techniques; and hunting bag composition. Positive associations between wild boar population density and ASF have been found (*4*), but contrary to earlier predictions, wild boar density does not seem to be a strictly limiting factor for persistence (*9*). The long-term availability of the virus in infected carcasses overtakes the expected density-dependent transmission pattern and enables the virus to persist despite any wild boar depopulation effort and the high mortality rate (*10*). Environmental persistence of the virus is favored by a cold and moist climate. In the ongoing outbreak, geography, ecology, meteorology, and wild boar demography all affect the epidemiology, and each contributes to the viability of the wild boar–habitat cycle. This association further suggests that ASF may persist in the habitat despite low availability of susceptible hosts.

Despite each epidemiologic cycle being independent, intercycle disease transmission will occasionally occur. Just as the intracycle spread in the domestic transmission cycle, such spread can be anthropogenic. Anthropogenic factors and intercycle spread from the domestic cycle to the wild boar–habitat transmission cycle seem to be causative factors for long-distance spread of ASF, thus contributing to sustaining and enlarging the geographic range of the wild boar–habitat transmission cycle in the ongoing epizootic.
